# Infectious mononucleosis complicated by transitory Epstein-Barr virus infection of T and natural killer cells

**DOI:** 10.1007/s12308-024-00595-6

**Published:** 2024-07-05

**Authors:** Yanlin Zhang, JianLan Xie, Yuanyuan Zheng, XiaoGe Zhou

**Affiliations:** Department of Pathology, Beijing Friendship Hospital, Capital Medical University, Beijing, 100050 People’s Republic of China

**Keywords:** EB virus, T/natural killer cells, Acute infection, NK/T-cell lymphoma

## Abstract

Epstein-Barr virus (EBV) typically infects B cells in infectious mononucleosis (IM), but a rare case shows EBV infection in T cells. Seven cases of lymphoproliferative disorder caused by EBV-positive cytotoxic T/natural killer (NK) cell proliferation in the lymph nodes, termed IM with transient EBV infection of T and NK cells (EBV + T/NK cells in IM), are reported here. The purpose of the study is to describe clinicopathological features of EBV + T/natural killer (NK) cells in IM of the lymph node. We retrospectively analysed seven cases of Chinese children and young people adults with EBV + T/NK cells in IM. We used morphological observation, immunohistochemical staining, EB virus in situ hybridisation detection, and analysis of T-cell receptor gene rearrangement. The patients were healthy prior to illness, experiencing sudden onset occurring in all the patients, with high fever as the first symptom, followed by lymphadenopathy and hepatosplenomegaly. Diagnosis occurred < 1.5 months of symptom onset. Most lymphocytes in lesions expressed CD3 and Granzyme B or TIA-1 and lacked CD5. CD56 was expressed in numerous cells in 5 of the 7 cases. EBV-encoded RNA (EBER) was detected in medium-to-large-sized cells (50–100 cells per cell/high-power field). T-cell receptor (TCR) gene rearrangement was seen in six cases, with monoclonal rearrangement in four cases. Treatment was conservative treatment but not chemotherapy. Four received anti-HLH therapy and others anti-inflammatory treatment. All patients survived with relapse after long-term clinical observation and follow-up. EBV + T/NK cells in IM can elicit malignant features that mimic T/NK-cell lymphoma pathologically and benign features mimicking IM clinically. These findings indicate that EBV + T/NK cells in IM could serve as valuable diagnosis. Additional clinical information, including age of onset (children and young people), nature of onset (sudden), disease course (short), symptoms (systemic), EBV infection status (acute), and lymph node involvement, is crucial for accurate diagnosis and prognostic evaluation.

Epstein-Barr virus (EBV) is a member of the herpes virus family with double-stranded DNA and was discovered by Epstein in 1964 through isolation from Burkitt’s lymphoma [1]. Generally, EBV infection occurs early in life and is usually asymptomatic. However, if primary EBV infection is delayed until adolescence and young adulthood, it can result in infectious mononucleosis (IM). IM is a self-limiting lymphoid disease with a benign clinical course [2], characterised by most EBV-infected cells being B cells with few T and natural killer (NK) cells [3]. Additionally, EBV is linked to malignancies, such as classical Hodgkin’s lymphoma, Burkitt’s lymphoma, T-cell lymphoma, and NK cell lymphoma. Recently, EBV has been associated with chronic active EBV infection (CAEBV) and T/NK cell lymphoproliferative disease (LPDs) [4].

Systemic EBV-positive T-cell lymphoma in children has been defined by the World Health Organisation (WHO) [5] as developing shortly after primary or acute EBV infection in previously healthy children and young adults or in the CAEBV setting. Historically, this process has been described using various terms, including fulminant EBV + T-cell LPD in childhood, sporadic fatal IM, fulminant hemophagocytic syndrome in children in Taiwan, fatal EBV-associated hemophagocytic syndrome in Japan, and severe CAEBV infection. Although it is a systemic disease, it mostly involves the liver and spleen, followed by the lymph nodes, bone marrow (BM), skin, and lungs. Patients with this disease usually die within days to months of diagnosis [6]. Ohshima et al. [7] proposed a new nomenclature to classify the pathological categories of EBV + T/NK-LPD in 2008, consisting of categories A1, A2, A3, and B. Category B is described as a monomorphic LPD with clonality and a fulminant course. In most studies, it has been described as a life-threatening disease.

EBV typically infects B cells in the IM, although a rare case has shown EBV infection in T cells [8]. We report seven cases of lymphoproliferative disorder caused by EBV-positive cytotoxic T/NK cell proliferation in the lymph nodes. We designated this condition as IM with transient EBV infection of T and NK cells (EBV + T/NK cells in IM). Notably, EBV + T/NK cells in the IM are easily misdiagnosed as T/NK cell lymphoma because they share similar histological and immunohistochemical features, including a cytotoxic T/NK cell phenotype and EBV positivity. However, based on its clinical characteristics, that is, spontaneous regression within a short time, EBV + T/NK cells in the IM should be recognised as reactive.

## Materials and methods

### Case selection and morphologic review

Information on seven cases with acute EBV infections of T/NK cells was obtained retrospectively from the files of the Department of Pathology, Beijing Friendship Hospital, Capital Medical University (Lymphoma Diagnosis and Research Center, Institute of Beijing Clinical Medicine). These cases were received for consultation during the period of June 2013 to June 2018. The final follow-up was on September 25, 2021. The common features among these seven cases of EBV + T/NK cells in IM were (1) T/NK cell predominant lymphoproliferation in the lymph node, (2) EBV infection primarily of cytotoxic T/NK cells, (3) EBV-positive cells of > 50/high-power field (HPF), (4) no radiotherapy or chemotherapy, and (6) long-term follow-up for a.

Clinical information included sex, age, duration of disease, initial symptoms, laboratory test results of blood and Epstein-Barr virus (EBV) detection, imaging examination of the liver and spleen, and follow-up results. Morphological observations included (1) lymph node structural change and degree of change, (2) complexity of T/NK cell type and composition, (3) presence of necrosis, and (4) lymphocyte atypia.

### Immunohistochemistry and EBER in situ hybridisation

Immunohistochemistry(IHC) staining was manually performed on formalin-fixed, paraffin-embedded (FFPE) tissues for immunophenotypic analysis. The MaxVision™ 2 kit (Cat. No. KIT-5910/5931) and monoclonal antibodies, against CD21, CD20, CD3, CD2, CD5, CD4, CD8, CD56, CD30, Granzyme B, TIA-1, PAX5, LMP1, and Ki-67, were provided by Maxin. Bio (provided by Maxin Bio, Fuzhou, CN, USA) and EBNA2 (provided by ABCOM) were used to detect all relevant antigens. Positive and negative controls were analysed according to the manufacturer’s instructions. The EBV Probe In Situ Hybridization Kit (Triplex International Biosciences (China) Co. Ltd., Fuzhou, China) was used to detect EBERs. Details of this procedure are described in our previous report [9]. The observers counted only definite EBV-positive cells, selecting fields with a high positivity rate. The total number of EBV-positive cells per high-power field (HPF) was recorded.

### Double staining

The immunohistochemical and EBER in situ hybridisation dual staining was conducted using a Leica Bond-III autostainer (Leica, Melbourne, Australia). Sections, 2-μm thick, were initially stained for EBER using DAB staining (brown); then, for CD3 (CD3; Maxin) and CD20 (L26; Maxin) was performed, the red staining was visualised after application of amino-ethylcarbazole (Bond Polymer Refine Red Detection kit, Leica) as chromogen [9].

### T-cell receptor gene clonality analysis

TCR gene rearrangement analysis was performed using the ‘Biomed-2’ primers (InVivoScribe Technologies, San Diego, CA, USA) [10]. DNA extracted from FFPE tissue samples utilised the TIANamp FFPE DNA Kit (DP331) (TIANGEN, Beijing, China). For the gene rearrangement assay, PCR reactions were conducted in a 25-μL volume containing 22.5 μL of master mix, 0.13 μL of AmpliTaq Gold DNA polymerase, and 100 ng of genomic DNA. The cycling profile comprised initial denaturation at 95 °C for 7 min, followed by 35 cycles of 95 °C for 45 s, 60 °C for 45 s, and 72 °C for 90 s; with a final extension at 72 °C for 10 min. Post-amplification, the PCR products were denatured at 94 °C for 5 min, followed by a quick chill to re-anneal the PCR products at 4 °C for at least 60 min and then electrophoresed on 6% polyacrylamide gels (Bio-Rad) in 1 × TBE buffer at 120 V for approximately 65 min. Gel was subsequently soaked for 20 min in 100 mL of 0.1 M NaCl solution containing 10 μL of 10 mg/mL Gel Red (Biotium, USA) and visualised under ultraviolet illumination for photography.

## Results

### Clinical features

**Fig. 1:**
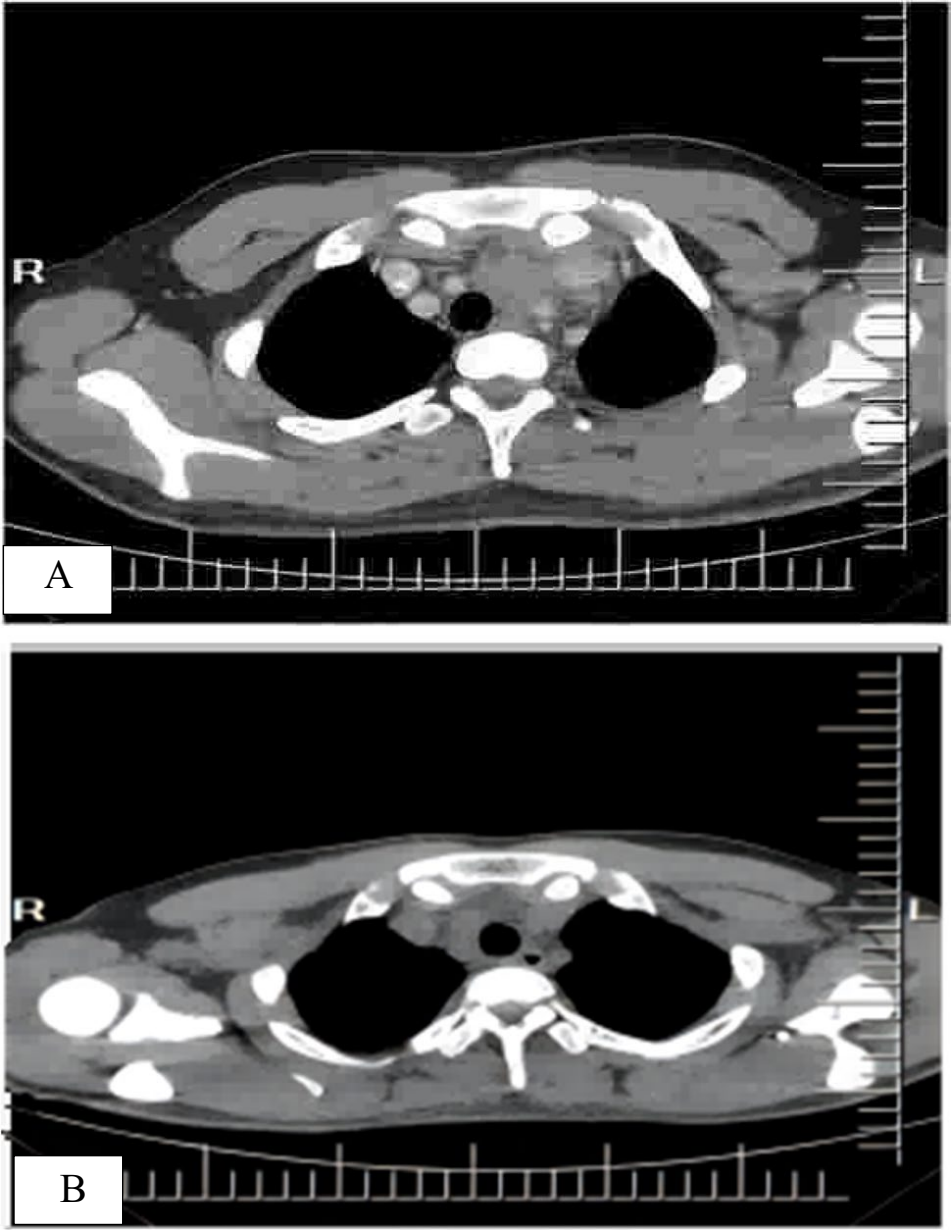
CT scan images are from the same patient (case 5). CT scan showing enlargement or fusion of lymph nodes, with no clear boundary and uniform density or necrosis (**A**). The follow-up CT scan showed no clear mass and enlarged lymph nodes (**B**)

The clinical characteristics of the seven patients are summarised in Table 1. Five patients were male and two were female. The age of the patients ranged from 10 months to 19 years, with a median age of 5.0 years. All the patients were children and young adults. They were healthy prior to the illness. Sudden onset was observed in all patients, with high fever as the first symptom, followed by lymphadenopathy and hepatosplenomegaly based on computed tomography (CT) (Fig. 1A, case 5). The disease course at diagnosis ranged from 1 to 1.5 months. Patients (patients 3 and 4) presented with pleural effusion and severe pneumonia. Laboratory examinations revealed reduced white blood cell counts at different levels in all the patients. Thrombocytopenia was observed in 4/7 patients, haemoglobin levels decreased in 6/7 patients, LDH levels increased in 4/7 patients, FERR ferritin (FERR) levels increased in 3/7 patients, and alanine aminotransferase (ALT) and/or aspartate aminotransferase (AST) levels increased in 5/7patients. Antibody testing for anti-EBV in the serum and detection of EBV DNA in the blood were available for six (patients 1, 2, 3, 4, 6, and 7). EBV-CA-IgM positivity was observed in three patients (patients 1, 2, and 7), and EBV-VCA-IgG positivity was found in six patients. These patients exhibited increased EBV DNA levels. The karyotype was detected in three patients, and no abnormalities were found (patients 1, 5, and 7). None of the patients had a history of hepatitis B or C infection, and no other immunodeficiency diseases were found. Four patients presented with hemophagocytic lymphohistiocytosis HLH (patients 2, 4, 6, and 7).

**Table 1:** Clinical data of 7 cases of IM complicated with EBV infection of T/NK cells

Case no	Sex/age	Course of disease	Clinical symptoms	Blood test	EBV detected in blood	Follow-up (month)
1	F/4 years	1 m	Fever, hepatosplenomegaly, lymphadenopathy	WBC 2.3 × 10^9^/L, HGB 78 g/L, PLT 56 × 10^9^/L, CRP 86.3 mg/L	EBV-CA-IgM( +) EBV-CA-IgG( +) EB-DNA 3.5 × 10^6^ copy/mL	36, NED
2	M/2 years	1 m	Fever, hepatosplenomegaly, lymphadenopathy	WBC 0.7 × 10^9^/L, HGB 71 g/L, PLT 17 × 10^9^/L, CRP 133 mg/L, LDH 893 U/L, FERR 10,440 ng/mL, ALT 103 U/L, AST 223 U/L	EBV-CA-IgM( +) EBV-CA-IgG( +) EB-DNA 1.6 × 10^4^ copy/mL	40, NED
3	M/19 years	1.5 m	Fever, hepatosplenomegaly, lymphadenopathy, pleural effusion, ascites, pneumonia	WBC 2.7 × 10^9^/L, HGB 112 g/L, PLT 119 × 10^9^/L	EBV-CA-IgM( −) EBV-CA-IgG( +) EB-DNA 4.3 × 10^5^ copy/mL	36, NED
4	M/3 years	1 m	Fever, hepatosplenomegaly, lymphadenopathy, pleural effusion, pneumonia	WBC 2 × 10^9^/L, HGB 98 g/L, PLT 140 × 10^9^/L CRP 111 mg/L, FERR 1179 ng/mL, LDH 695 U/L, AST 51.8 U/L	EBV-CA-IgM( −) EBV-CA-IgG( +) EB-DNA 2.01 × 10^4^ copy/mL	38, NED
5	M/10 months	1 m	Fever, hepatosplenomegaly, lymphadenopathy	WBC 3.76 × 10^9^/L, HGB 106 g/L, PLT 256.4 × 10^9^/L, CRP 3 mg/L, LDH 754 U/L, AST 61.6 U/L	NA	84, NED
6	F/1 years	1 m	Fever, hepatosplenomegaly, lymphadenopathy	WBC 1.99 × 10^9^/L, HGB 102 g/L, PLT 63 × 10^9^/L, ALT 206 U/L, AST 286 U/L, LDH 1670 U/L, CRP 1.43 mg/L	EBV-CA-IgM( −) EBV-CA-IgG( +) EB-DNA 1.09 × 10^4^ copy/mL	70, NED
7	M/5 years	1.5 m	Fever, hepatosplenomegaly, lymphadenopathy	WBC 2.32 × 10^9^/L, HGB 94 g/L, PLT 75 × 10^9^/L, ALT 370 U/L, AST 2392 U/L, FERR 1495.3 ng/mL, CRP 21 mg/L	EBV-CA-IgM( +) EBV-CA-IgG( +) EB-DNA 4.0 × 10^6^ copy/mL	37, NED

*NED* no evidence of disease, *WBC* white blood cell, *HGB* haemoglobin, *PLT* platelet, *CRP* C-reactive protein, *LDH* lactate dehydrogenase, *FERR* ferritin, *ALT* alanine aminotransferase, *AST* aspartate aminotransferase, *MOF* multiple organ failure, *NA* not available, ( −) EBV antibodies were not detected, ( +) EBV antibody titre higher than the reference value

### Morphological features

**Fig. 2:**
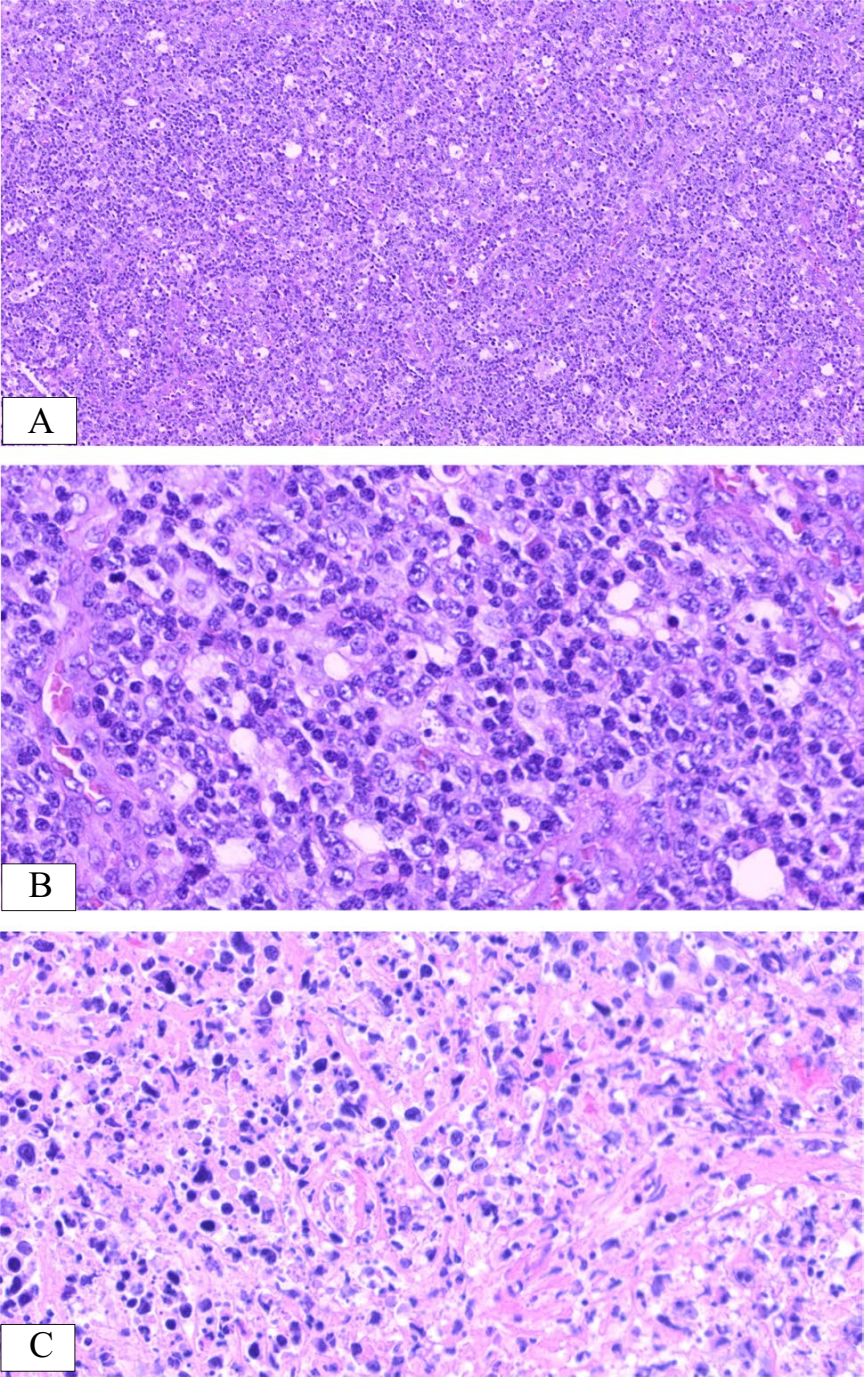
Histologic presentations of EBV + T/NK cells in IM from case 4. Lymph node structure damage (H&E, × 100) (**A**). Pleomorphic large atypical cells with irregularly folded nuclei (H&E, × 400) (**B**). Coagulative necrosis displaying multifocality (H&E, × 400) (**C**)

The histopathological changes in the seven patients are summarised in Table 2. The lymph nodes were removed from all samples for biopsy. The architecture was mostly destroyed, and a portion of the lesions involved the peripheral tissue of the node (Fig. 2A). The cortex and medulla of the node were indistinct, and a few residual follicles and subcapsular sinuses that existed were challenging to discern. The cell compositions were diverse. Furthermore, neutrophilic granulocytes, eosinophils, and plasma cells were identified at high magnification. The lesions exhibited numerous infiltrating lymphocytes with medium to large sizes, round to mildly irregular nuclei, inconspicuous or small nucleoli, and dispersed chromatin. Mitotic figures were readily detected, and most cells exhibited significant atypia (Fig. 2B). The lesions were diffuse, with focal-to-extensive coagulative necrosis (Fig. 2C). Additionally, lymphocyte infiltration into the vascular walls was observed.

**Table 2:** Morphology, antigen expression, EBER in situ hybridisation, and TCR gene rearrangements’ results of 7 cases of IM complicated with EBV infection of T/NK cells

Case no	Structure destruction	Cell components	Atypia	Coagulative necrosis	CD21	CD3	CD5	CD56	TIA-1	Granzyme B	LMP1	PAX5	CD4	CD8	EBNA2	CD30	Ki-67	EBER (HPF)	TCR
1	Yes	L	H	Yes	−	+ + +	+	+	+	+	−	−	− / +	+ +	+	+	60%	50 ~ 100	M
2	Yes	M	M	No	−	+ + +	−	−	+	NA	−	−	− / +	− / +	+	−	40%	> 100	M
3	Yes	L	H	Yes	−	+ + +	+	+	NA	+	+	−	− / +	+ +	−	+	60%	50 ~ 100	NA
4	Yes	L	H	Yes	−	+ +	−	+	+	NA	−	−	−	+ +	+	−	70%	> 100	P
5	Yes	L	H	Yes	−	+ + +	+	−	NA	+	+	−	− / +	+ + +	NA	−	70%	> 100	M
6	Yes	L	H	Yes	−	+ + +	−	+	+	+	−	−	−	+ + +	NA	+	70%	> 100	M
7	Yes	L	H	No	−	+ + +	+	+	+	+	−	−	−	− / +	+	+	60%	> 100	P

*L* predominantly composed of large cells, *M* predominantly composed of medium cells, *S* predominantly composed of small cells, *H* high atypia, *M* moderate atypia, *NA* not available, *M* monoclonal rearrangement, *P* polyclonal rearrangement

− , EBV-negative in EBV infection cells; − / + , positive less than 10% EBV infection cells; + , positive about 11–30% EBV infection cells; +  + , positive about 31–50% EBV infection cells; +  +  + , positive for more than 51% of EBV-infected cells

### Immunohistochemical and in situ hybridisation findings

**Fig. 3:**
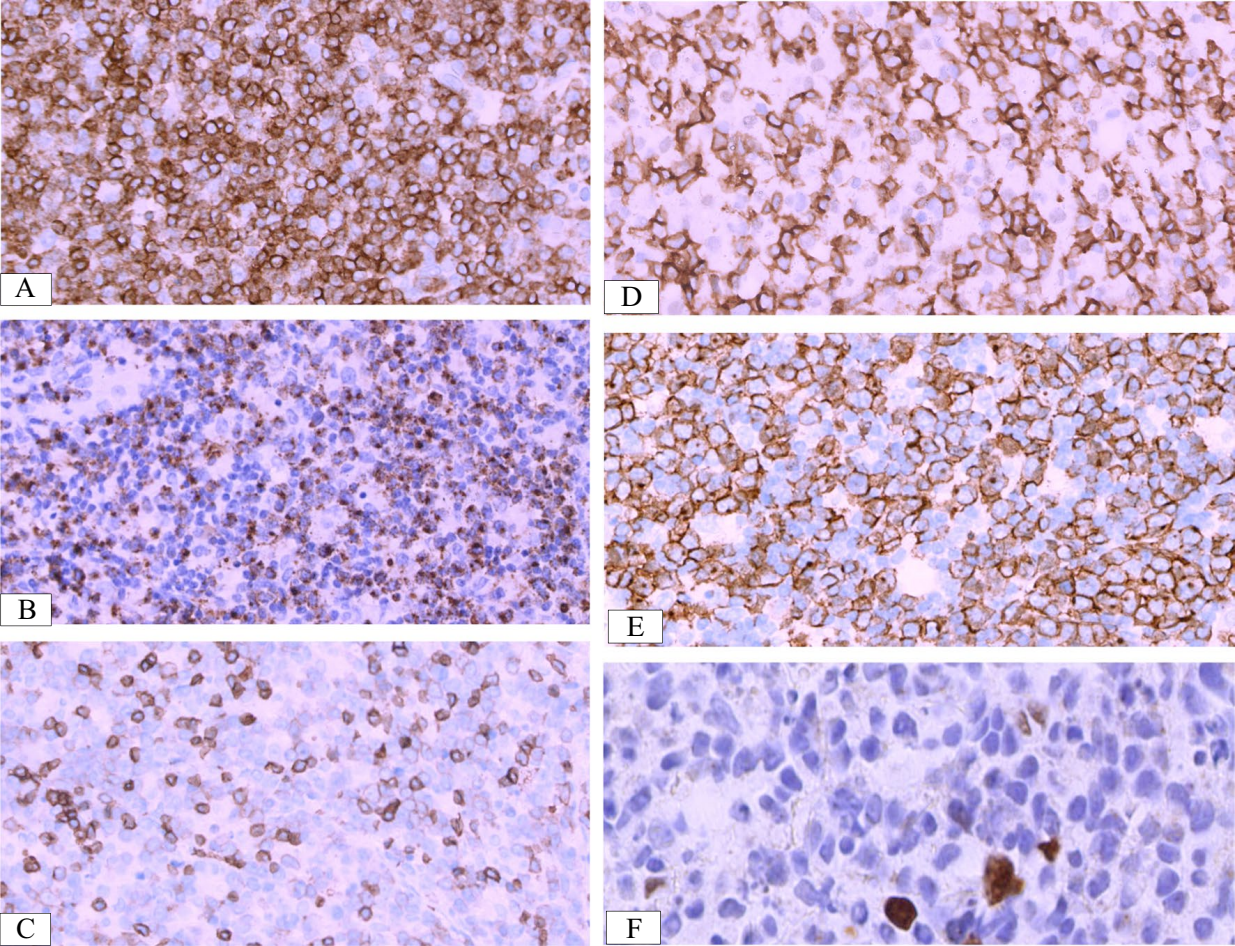
Immunohistochemical features of EBV + T/NK cells from case 4. Atypical cells exhibiting cytoplasmic CD3 (IHC, × 400) (**A**). Pleomorphic cells were positive for Granzyme B (IHC, × 400) (**B**). CD5-positive cells less than CD3 (IHC, × 400) (**C**). Atypical cells showing strong CD56 staining (IHC, × 400) (**D**). Almost all cells showed strong granular staining for CD8 (IHC, × 400) (**E**). EBNA2 showed scattered positive in EBER + cells (IHC, × 800) (**F**)

**Fig. 4:**
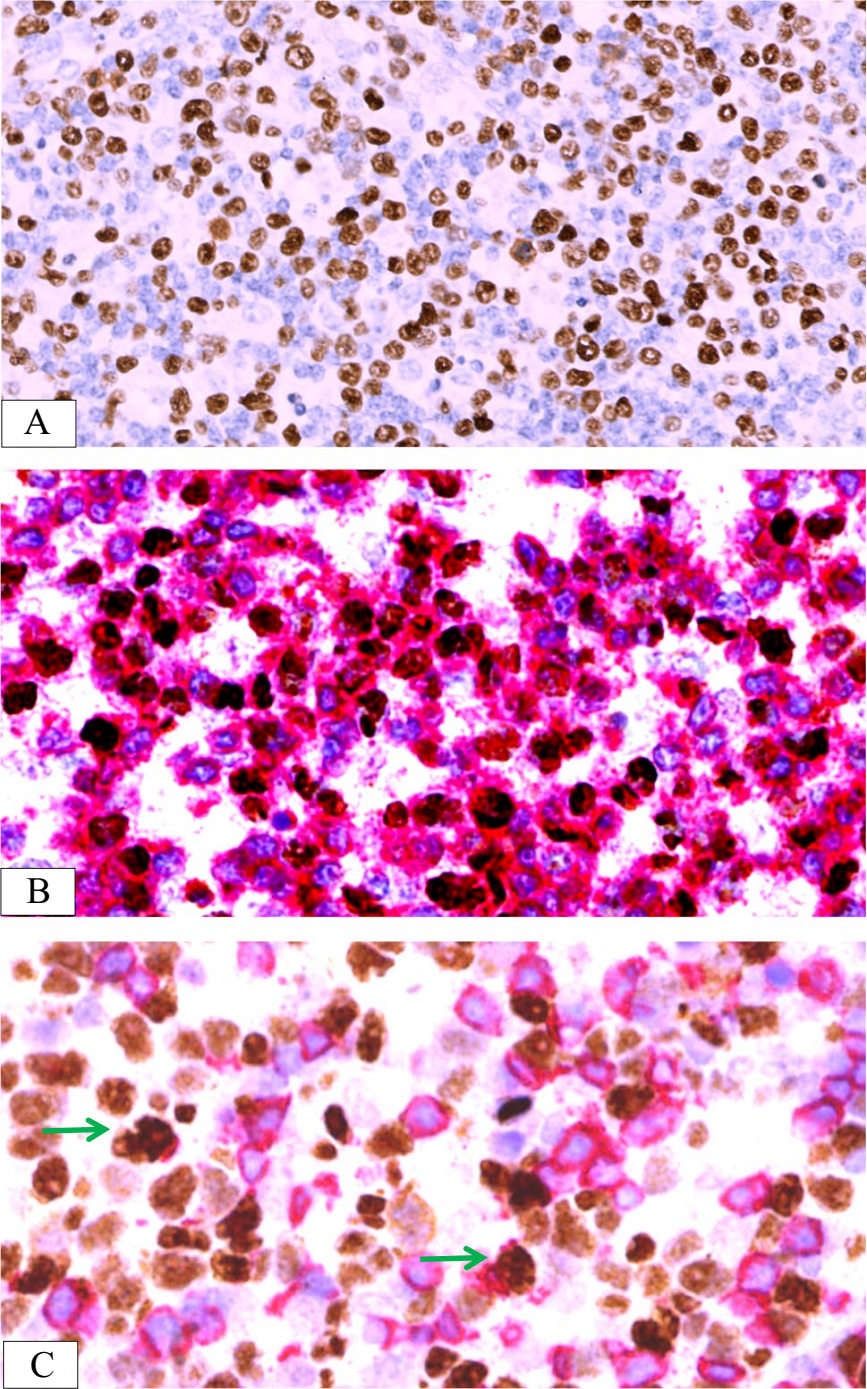
EBER and double staining of EBV + cells from case 2. In situ hybridisation of EBV-encoded RNA (EBER). In these lesions, almost all irregular cells showed nuclear labelling (IHC, × 400) (**A**). EBER-positive cells (brown) with CD3 expression (red) (× 800) (**B**). EBER-positive cells (brown) were scattered among CD20 + cells (red) (× 800) (**C**, green arrow)

The immune phenotype and EBV infection characteristics of the seven patients are summarised in Table 2; the majority of lymphocytes in the lesion expressed CD3 and TIA-1 or Granzyme B (Fig. 3A, B) but not CD5 (Fig. 3C) in all cases. CD56 expression in the same area was seen in five cases (Fig. 3D). Only a few scattered lymphocytes express CD20. The number of CD8-positive cells (Fig. 3E) was higher than that of CD4-positive cells in six cases. One patient (case 2) had a reduced number of CD4- and CD8-positive cells, and the degree of reduction was similar for both cell types. Ki-67 staining was positive in more than 50% of the cells, except in case 2, where only 40% positivity was observed. The larger scattered cells were CD30-positive in four cases (cases 1, 3, 6, and 7) and LMP1-positive cells in two cases (cases 3 and 5). EBNA2-positive cells were observed in four cases (cases 1, 2, 4, and 7; Fig. 3F). EBER in situ hybridisation revealed a positive signal localisation in the nucleus. EBER-positive cells were observed in the CD3 + /CD56 + areas in five cases, while the other two cells were observed in the CD3 + /CD56 − areas. No EBER-positive cells were observed in the PAX5 + areas in all cases. More than 50 EBER-positive cells per high-power field (> 50/HPF) were observed, with varying cell sizes (Fig. 4A). Double staining for EBER/CD3 and EBER/CD20 showed that most EBER-positive cells were located in the CD3 + areas in all cases (Fig. 4B, C), with only scattered EBER-positive cells found in CD20 + cells.

### PCR for TCR gene rearrangements

T-cell clonality analysis by BIOMED-2 PCR revealed that four cases (cases 1, 2, 5, and 6) presented a monoclonal rearrangement, but the other two cases were not tested (Table 2).

### Follow-up

Follow-up data were available for all seven patients. Four patients (cases 2, 4, 6, and 7) underwent anti-hemophagocytic lymphohistiocytosis (HLH) therapy, whereas the others received anti-inflammatory treatment. All patients survived. The follow-up duration since diagnosis was 36–84 months (mean duration of 48.7 months). Follow-up CT showed no clear mass or enlarged lymph nodes (Fig. 1B, case 5). The symptoms of all patients were relieved, with no fever, and the lymph node, liver, spleen, and blood test results were within the normal range.

## Discussion

EBV-positive lymphoproliferative diseases encompass a range of conditions, including both reactive and malignant disorders. In this study, we examined seven patients diagnosed with EBV + T/NK cells in the IM. All patients survived and are currently under long-term clinical observation and follow-up. It is important to recognise this disease and differentiate it from NK/T-cell lymphoma, systemic EBV + T-cell lymphoma in children, and HLH presenting with extensive infection of T and NK cells. It is important to recognise this complication, and the differential diagnoses include NK/T-cell lymphoma, systemic EBV + T-cell lymphoma in childhood, and HLH.

IM is an acute disease, primarily caused by EBV infection, predominantly affecting B cells, although rare cases involve T cells [11] and NK cells. It typically manifests with high fever, pharyngitis, and cervical lymphadenopathy and often resolves spontaneously. Microscopically, lymph nodes show obvious paracortical expansion and focal destruction of the lymph node architecture. The infiltrating cells are heterogeneous and include small and large lymphocytes, immunoblasts, histiocytes, and variable numbers of plasma cells and eosinophils [12]. The histological features of IM are so varied that they are sometimes misdiagnosed as malignant lymphoma, particularly in cases with numerous large immunoblasts. The clinical symptoms and prognosis described in our present cases were similar to those of IM, except for differences in the types of EBV-infected cells and certain morphological characteristics. While there were two reports describing an IM patient with atypical T-cell proliferation in the nasopharynx, most lymphoid cells were also positive for CD2, CD3, CD5, CD7, and without loss of T-cell antigen [8, 9]. In the present case, the clinical manifestations and blood EBV antibody test results of some patients were consistent with IM; however, excessive EBV infection of T/NK cells has raised concerns about lymphoma. The staining of EBNA2 helps determine the type of EBV infection in pathology, thereby avoiding overdiagnosis of lymphoma. In the IM, T and NK cells may occur, and these might even be monoclonal [13].

NK/T-cell lymphoma occurs mostly in adults, is invasive, progresses rapidly, and almost always exhibits an extranodal presentation. The nasal cavity, nasopharynx, paranasal sinuses, and palate are most commonly involved, with the nasal cavity being the prototypic site of involvement [5]. Some cases may also be accompanied by secondary lymph node involvement [14–16]. Isolated nodal involvement in NK/T-cell lymphoma is extremely rare, and only a few cases have been described in published work [17–21]. NK/T-cell lymphoma is highly aggressive, with short survival times and poor response to therapy; therefore, the clinical outcomes are dismal. Differentiating between EBV + T/NK cells in IM and NKTL based on morphology and immunophenotype is difficult. However, NK/T-cell lymphoma often lacks systematic clinical manifestations at onset and progresses rapidly, causing significant damage to adjacent tissue structures. At the same time, we observed positive EBER staining in cells of varying sizes in our cases, suggesting that EBV infection affected cells at different stages of differentiation. The self-limiting characteristic of EBV + T/NK cells in IM underscores its clinical significance. When managing such conditions, careful considerations and selection of treatment strategies are paramount.

Systemic EBV + T-lymphoma in children, according to the WHO classification [5] and Category B classification by Ohshima et al. [7], develops shortly after primary or acute EBV infection and is accompanied by an aggressive clinical course and atypical lymphoid cell infiltration, with most reported cases exhibiting a monoclonal pattern of T-cell proliferation, progressing toward multiple organ failure, sepsis, and sudden death [22, 23]. These malignant tumour features overlapped with those of our present cases in terms of clinical manifestations, pathological morphology, immune phenotype, and clone detection; however, the present patients achieved remission or recovered without relapse during long-term clinical observation. A similar group of cases has been reported [24]; however, the patients died quickly, which differs from our study results. The presence of an abnormal karyotype favours neoplastic conditions and is helpful in identifying systemic EBV + T-lymphoma in children [25]. In the present case, karyotype testing of the three patients showed no abnormalities. The response to HLH treatment is one of the most important parameters for distinguishing IM to Systemic EBV + T-cell lymphoma in childhood.

In the present cases, four patients mimicked EBV-associated HLH, which was described as young age and EBV + T/NK cell proliferation with variable clinical findings, including high fever and splenomegaly, cytopenia, and liver dysfunction, and was accompanied by histological evidence of hemophagocytosis, which causes extremely high serum levels of ferritin, lactate dehydrogenase, and soluble CD25, serological tests, or the detection of EBV DNA or RNA from tissues [26]. After anti-HLH treatment, 4 patients achieved complete remission. The other three patients did not exhibit HLH. In addition to observation and follow-up, symptom relief was achieved without any special treatment. EBV-associated hemophagocytic lymphohistiocytosis (HLH), a hyperinflammatory syndrome induced by a dysregulated immune reaction secondary to EBV infection, is difficult to differentiate in childhood. Clonal EBV-infected T cells have been demonstrated in some cases [13], and patients typically respond to treatment, which can be limited in some cases, with complete recovery following acute disease. Therefore, EBV + T/NK cells in IM can be considered a clinical manifestation of HLH, and the prognosis is generally good after treatment.

Primary EBV-positive nodal T-cell or NK-cell lymphomas have been reported [5, 20, 27]. These lymphomas typically present with lymphadenopathy with or without extranodal involvement, advanced-stage disease, and B symptoms. They exhibit a monomorphic pattern of infiltration and lack of angiodestruction and necrosis seen in extranodal NK/T-cell lymphomas. They are more common in elderly patients or patients with immune deficiency and have a dismal prognosis. Although its immunophenotype is similar to that of EBV + T/NK cells in the IM, it often occurs in the lymph nodes. However, it is more common in elderly patients and has a poor prognosis, which differs from the present case.

It should be noted that a positive monoclonal population does not necessarily predict malignant behaviour because similar populations can be observed under reactive conditions [28–31]. We identified a clonal T-cell population in four cases (cases 1, 2, 5, and 6). Clonality does not necessarily indicate malignancy, and our results showed that monoclonal T-cell populations are visible in patients with primary EBV infection, consistent with previous reports [13]. Furthermore, the prevention of serious complications such as multiple organ failure, hemophagocytic syndrome, disseminated intravascular coagulation, and sepsis is very important.

It is pertinent to perform a detailed laboratory examination of either EBV load in the serum or expression of EBV in situ to evaluate cases with a sudden onset course. In the present case, serological studies were incomplete or had not been performed. EBV-CA-IgM positivity was observed in three patients (patients 1, 2, and 7), EBV-CA-IgG positivity was observed in six patients, and six patients showed increased EBV DNA. EBV serology in such cases may be clinically misleading, as it may not conclusively indicate acute primary or active infection. However, the precipitous onset of symptoms in previously healthy young individuals is along with biopsy samples showing infiltration of numerous EBER-positive and scattered EBNA2-positive cells suggesting an acute process. In particular, CD8-positive cells increased, suggesting primary EBV infection. EBNA2 (Epstein-Barr virus nuclear antigens 2) is a gene product expressed during latent EBV infection. As a key transcription factor, it regulates the expression of viruses and many genes in cells during viral infection of lymphocytes [11]. Furthermore, EBNA-2 was expressed in four cases with scattered positive nuclei, three of which had increased anti-EBV VCA IgM antibodies. EBNA2 is a useful marker expressed in patients who have a primary immune response or are immunocompromised; IM patients usually have EBNA2-positive cells [9]. Additionally, in the present cases, the indices of liver function and other markers of illness severity in EBV infection were either incomplete or had not been evaluated previously. Therefore, patients with a high viral load in their blood and particularly intense expression of EBV in their vital organs require more intense clinical supervision.

EBV + T/NK cells in the IM can elicit both pathologically malignant and clinically benign features. Thus, detailed clinical information including age at onset (children and young people), nature of onset (sudden), disease course (short), symptoms (systemic), EBV infection status (acute), and lymph node involvement is crucial for the diagnosis and prognostic evaluation of this disease.

## Data Availability

The authors confirm that the data supporting the findings of this study are available within the article [and/or its supplementary materials].
